# Atopic dermatitis in cats and dogs: a difficult disease for animals and owners

**DOI:** 10.1186/s13601-018-0228-5

**Published:** 2018-10-05

**Authors:** Natalie Katharina Yvonne Gedon, Ralf Steffen Mueller

**Affiliations:** Small Animal Medicine Clinic, Centre for Clinical Veterinary Medicine, Ludwig Maximilian University, Veterinaerstraße 13, 80539 Munich, Germany

**Keywords:** Allergy, Canine, Feline, Atopy-like dermatitis, Adverse food reaction, IL-31, Lokivetmab, Immunotherapy

## Abstract

The purpose of this review article is to give an overview of atopic dermatitis in companion animals and of recent developments including knowledge on immunological background, novel treatment options and difficulties in disease management. The prevalence of hypersensitivities seems to be increasing. The pathogenetic mechanisms are not fully understood, yet multiple gene abnormalities and altered immunological processes are involved. In dogs and cats, the diagnosis of atopic dermatitis is based on history, clinical examination and exclusion of other differential diagnoses. Intradermal testing or testing for serum allergen-specific Immunoglobulin E is only used to identify allergens for inclusion in the extract for allergen immunotherapy. Symptomatic therapy includes glucocorticoids, ciclosporin, essential fatty acids and antihistamines. A selective janus kinase 1 inhibitor and a caninized monoclonal interleukin-31 antibody are the newest options for symptomatic treatment, although longterm effects still need to be assessed. The chronic and often severe nature of the disease, the costly diagnostic workup, frequent clinical flares and lifelong treatment are challenging for owners, pets and veterinarians. Patience and excellent communication skills are needed to achieve a good owner compliance and satisfactory clinical outcome for the animal.

## Background

Atopic dermatitis (AD) is a common skin disease in dogs and cats. Its clinical, immunological, histological and pathological features in dogs are so similar to the human counterpart, that canine atopic dermatitis has been suggested as an animal model for human AD [1, 2]. In Table 1 some of the similarities and differences are summarized. Much less is known on the pathogenesis in cats, but the clinical findings are different to those seen in humans and dogs.

**Table 1:** Similarities and differences of AD in dogs and humans

	Dogs	Humans
Pathogenesis	Th2 immune response Skin barrier damage Allergic inflammation [18, 19, 153]	Th2 immune response Skin barrier damage Allergic inflammation [154]
IL-4 and IL-13	Pruritus, acute inflammation [155]	Pruritus, acute inflammation [156, 157]
Periostin (PO) expression	Increased expression, related to the chronicity of skin lesions [158]	Increased expression, related to the chronicity of skin lesions [159, 160]
Histologic pattern	Spongiotic, hyperplastic dermatitis with mononuclear infiltrate; predominantely T-lymphocytes [153, 161]	Spongiotic, hyperplastic dermatitis with mononuclear infiltrate; predominantely T-lymphocytes [162, 163]
Dysbiosis	Reduced microbiome diversity [164] and fungal dysbiosis [165]	Reduced microbiome diversity and fungal dysbiosis [166]
Clinical signs	Eczematous skin lesions with no progression of clinical signs e.g. no development of asthma [2, 44]	Atopic march
Allergy testing	Intradermal testing without high risk of anaphylactic reactions [69]	Skin prick testing
Immunotherapy	Accelerated immunotherapy without increased risk for anaphylactic reactions [76, 78, 79]	Standard AIT

## Canine atopic dermatitis

Canine AD is a multifactorial disease process. It is defined as a “genetically predisposed inflammatory and pruritic allergic skin disease often associated with a production of immunoglobulin (Ig) E against environmental allergens” [3]. The estimated prevalence of AD in the dog is approximately 10–15% [4]. Although the pathogenesis is not completely understood, there is evidence for genetic abnormalities, an altered immune system with cutaneous inflammation and a skin barrier defect [5, 6].

### Genetic background

Multiple gene expressions involved in skin barrier function and cutaneous inflammation have been described as down- or upregulated in the skin of privately owned atopic dogs [7–9] as well as in a canine model of AD [10]. In the latter study, 361 genes relevant for inflammation, wound healing or immune response processes showed an increased expression, whereas 226 genes associated with differentiation and skin barrier function showed decreased mRNA concentrations in allergen-treated skin of sensitized dogs [10]. In atopic German shepherds a significant association with chromosome 27 was determined, especially with genes that had a connection to plakophilin 2 production [11]. Plakophilin 2 is an important structural protein, which is expressed in epithelial and immune cells [11, 12]. The predisposition of German shepherds for AD is likely due to a risk haplotype in combination with multiple variants resulting in a changed expression of the plakophilin 2 gene and nearby genes [11]. In the United Kingdom the risk of Labrador and Golden retrievers to develop AD was almost 50% due to the genetic background [13, 14]. Multiple breeds including Boxer, Westhighland White Terrier, French bulldog, Bullterrier, American cocker spaniel, English springer spaniel, Poodle, Chinese Sharpei, Dachshund, Collie, Miniature schnauzer, Lhasa apso, Pug and Rhodesian ridgeback are also predisposed [15, 16] and breed predispositions vary with geographic location [17].

### Immunologic alterations

In acute lesions, allergic inflammation triggers the release of cytokines such as interleukin (IL-) 4 and IL-13, which induce a T helper 2 (Th2) response [1, 18, 19]. In more chronic skin lesions, CD4+ and CD8+ skin-associated T lymphocytes additionally stimulate the production of various cytokines such as IL-13, IL-22 and IFN-γ [20]. Recent findings on cytokines and specific cell types in atopic dogs are listed in Table 2.

**Table 2:** Recent findings on T cells and cytokines in canine atopic dermatitis

Cytokine/cell	Function
IL-31	Important role in atopic pruritus [167]. Its serum concentration correlates with the severity of active skin lesions [168]
IL-13	Induces production of PO in keratinocytes and fibroblasts, associated with chronicity of skin lesions and their deterioration [1]
IL-25	Increased in PO-stimulated keratinocytes [1], clinical relevance unclear. In a murine asthma model relevant for Th2-mediated immunity, contributes to a decreased epidermal barrier function in human AD [169–171]
IL-33	Upregulated in chronic lesional skin, similar to atopic humans [172]
CD 34+ cells	Increase in peripheral blood, unclear clinical relevance [173]
CD4+ CD25+ FoxP3+ cells	Significantly higher percentage in peripheral blood and correlated with severity of AD [174]

### Skin barrier defects

According to the “outside-in” theory an impaired epidermis leads to an increased allergen penetration and hence a higher allergen exposure of epidermal immune cells [21]. This skin barrier defect may be due to decreased filaggrin concentrations [22]. Caspase 14 is involved in the breakdown of filaggrin into natural moisturizing factors such as free amino acids and small peptides and altered concentrations might influence the skin barrier function and hydration of the stratum corneum [23, 24]. Conflicting results regarding the filaggrin metabolism in atopic dogs have been published with lower [22] and higher caspase 14 concentrations [24]. Changes in the ceramide composition of lesional canine atopic skin have been described [25, 26] contributing to disorganisation of the lipid envelope and hence disruption of the epidermal barrier. Ceramide profiles of atopic dog skin contained lower amounts of CER [EOS], CER [EOP] and CER [NP] [27], similar to what is seen in humans. A decreased relative content of ceramides in atopic dogs might be one reason for the increased transepithelial water loss observed in both lesional and non-lesional skin [28]. Moreover, house dust mite allergens can alter the expression and possibly also the function of corneodesmosomal and tight junction proteins through proteolytic digestion and/or allergic inflammation, facilitating a higher allergen penetration through the epidermis [29].

## Feline atopy-like dermatitis

The function of IgE in the cat is not completely clarified, consequently the term “feline atopic dermatitis” is not ideal [30, 31], but rather it is refered to as “feline atopy-like dermatitis”. The pathogenesis of feline atopy-like dermatitis is not completely elucidated. Data on genetic alterations and skin barrier abnormalities as reported in human and canine AD are rare.

### Genetic background

In a large study evaluating allergic cats, pure-bred cats were overrepresented in the group of cats with atopy-like dermatitis compared to cats with flea allergy, but the study lacked a non-allergic control group [32]. In this study, Abyssinians were only affected by atopy-like dermatitis and not flea allergy. A predisposition for Devon rex, Abyssinian and domestic shorthaired cats was reported in another study [33]. A case report of three littermates with clinical signs and history consistent with atopy was described implying a heritable factor [34], however more detailed genetic studies are lacking [31].

### Immunologic and skin barrier alterations

In cats, histopathologic features of atopy-like dermatitis include perivascular to diffuse dermal infiltration of T lymphocytes, activated antigen presenting cells, eosinophils, macrophages and high numbers of mast cells [35]. A significant increase of CD4+ T cells, IL-4 and CD1a+ dentritic cells was found in the skin of cats with atopy-like dermatitis, pointing to a Th2-mediated immune dysfunction [33, 36], although cytokine pathways have not been investigated [37]. Comparable to humans and dogs [38] a fungal dysbiosis was found with next generation sequencing of skin swabs taken from healthy and allergic cats [39]. Skin hydration as a measure of the skin barrier did not always correlate with clinical scoring indicating that a barrier defect may not be as relevant in cats [40].

## Practical approach

### Clinical features

The following three main allergy categories can be distinguished in cats and dogs: flea (and other insect bite) hypersensitivities, cutaneous adverse food reaction (AFR) and AD due to environmental allergens. The clinical signs in the atopic dog are mostly distinct when compared to the atopic cat. A short overview of the main clinical features, diagnosis and treatment options in companion animals is given in Table 3.

**Table 3:** Clinical features, diagnosis and treatments of atopic dermatitis for small animals

	Dog	References	Cat	References
Age	Commonly 6 months to 3 years	[41]	Commonly < 3 years	[31, 32]
Clinical symptoms	Pruritus		Eosinophilic granuloma complex (indolent eosinophilic ulcer, eosinophilic granulomas, eosinophilic plaques)	[32, 46, 47]
	Inflammation (Erythema, self-induced alopecia, excoriation) secondary infection	[41, 42]	Head and neck pruritus	
			Miliary dermatitis	
			Self-induced alopecia	
Affected body part	Ear pinnae, axillae, ventral abdomen, extremities, paws, inguinal, lips, perianal region	[42, 43]	Head, mouth, neck, abdomen, trunk	
Diagnosis	Exclusion diagnosis (rule out differential diagnosis, compatible history and clinical signs		Exclusion diagnosis (rule out differential diagnosis, compatible history and clinical signs	
Therapy	Allergen contact avoidance	[71]	Allergen contact avoidance	
	Specific targeted: Allergen-specific immunotherapy	[70, 72–79, 81, 82]	Specific targeted: Allergen specific immunotherapy	[33]
	Untargeted, symptomatic:		Untargeted, symptomatic:	
	Glucocorticoids	[85]	Glucocorticoids	
	Ciclosporin	[86, 87, 89]	Ciclosporin	[88, 90, 91]
	Oclacitinib	[92–95]	Oclacitinib	[96]
	Lokivetmab	[83, 84]		
	Antihistamines	[97–100, 103–105]	Antihistamines	[33, 106]
	Topical:		Topical:	
	Shampoos	[113, 114]		[110]
	Hydrocortisone-aceponate	[108, 109]	Hydrocortisone-aceponate	
	Tacrolimus	[111, 112]		
Supportive dietary interventions	Essential fatty acids	[116–119]	Essential fatty acids	[115]
	Probiotics	[124, 125]		
	Cholecalciferol	[129]		

### Clinical features of canine AD

In dogs, clinical signs of an environmental allergy mainly develop between 6 months and 3 years of age [41]. Erythema is a primary lesion of canine AD; pruritus and inflammation can result in self-induced alopecia, excoriation and secondary infections with papules, pustules and crusts [41, 42]. Axillae, ventral abdomen, distal extremities, inner pinnae and periocular, perioral and perianal regions are commonly affected [42]. Otitis externa is present in half of the dogs with AD. Predilection sites differ from breed to breed [43]. Even though dogs can have multiple target organs for hypersensitivities (including gut and respiratory) [44], the contact with environmental allergens predominantly induces skin lesions in this species [45]. There is no evidence for the progression of initially exclusive cutaneous lesions to respiratory signs and systemic hypersensitivities comparable to the “atopic march” in humans [44]. In contrast to the cat, clinical examination in the dog frequently provides clues on the pathogenesis of the pruritus as to the presence of flea bite hypersensitivity versus environmentally-induced atopy or AFR. The former is characterized by pruritus focused on the dorsal lumbosacral area, ventral abdomen, tailbase and thighs.

### Clinical features of feline atopy-like dermatitis

The manifestation of specific cutaneous reaction patterns [46] can indicate an allergic primary cause in cats. These involve head and neck pruritus, miliary dermatitis characterised by small crusted papules, self-induced alopecia without any other clinical lesions and eosinophilic lesions such as eosinophilic indolent ulcers, eosinophilic granulomas and eosinophilic plaques [32, 47]. In rare cases, untypical AD symptoms such as plasma-cell pododermatitis, seborrhoea, ceruminous otitis, facial erythema and exfoliative dermatitis were reported [31, 48]. Additionally noncutaneous signs such as sneezing, coughing, conjunctivitis, diarrhoea or vomiting can be presented in affected cats [32]. The disease onset can vary, but commonly it is under 3 years [31, 32], whereas the mean age for AFR is slightly higher (approximately 4–5 years) with a range from 3 months to 11 years [48]. In contrast to the dog, flea-bite hypersensitivity and environmentally induced and AFR look much more similar in the cat [32].

### Diagnosis

A differential diagnosis of AD is based on age of onset, breed and clinical signs. Other differential diagnoses such as ectoparasites and flea bite hypersensitivity must be ruled out by a consequent ectoparasite control. There is no single test differentiating the atopic from the non-atopic dog or cat [49].

It is not possible to distinguish clinical signs of AD caused by perennial environmental allergens from AFR [16, 50, 51]. Hence an elimination diet followed by a provocation with the original food should be performed in any dog or cat with non-seasonal AD [52], particularly those with a long history of pruritus and/or gastrointestinal signs [51, 53]. A diet length of 6–8 weeks is recommended, as 90% of the dogs with AFR show some improvement during this time period [54]. Every food can potentially result in an AFR [55]. The most common reported causative allergens for canine AFR are beef, dairy products, chicken, wheat, and lamb [56]. However, soy, corn, egg, pork, fish and rice have also been reported as offending allergens [56]. The food sources most frequently causing AFR in cats were beef, fish, and chicken [58]. Wheat, corn, dairy products, lamb, egg, barley and rabbit were also reported as offending allergens in individual cats. The selection of appropriate protein and carbohydrate sources for an elimination diet can be challenging. It is important to use a protein and carbohydrate source, which the dog or cat has never received before [52], thus a detailed food history needs to be obtained by the veterinarian. Multiple studies have shown that various commercial special diets with only one protein source on their label were contaminated and contained substances not listed on the label [57–60]. Highly hydrolysed food is an alternative, but some dogs allergic to chicken also react to diets containing hydrolysed chicken protein [61]. Therefore a home cooked diet by the owner is considered as diagnostic gold standard [52], where instead of commercial dry or canned food the owner purchases one type of meat and one carbohydrate source and prepares those him-/herself for the pet. As cats are obligate carnivores, the use of a carbohydrate source is optional in the short term and indeed may reduce palatability. Currently there is no reliable alternative test for diagnosing food allergy [62]. There is only poor correlation between IgE- and IgG-antibodies in the serum and clinical food reactions [53, 63]. A patch test can be used for the selection of the elimination diet food source if the food history is unknown. This test has a poor positive predictability, but a high negative predictability [53]. A lymphocyte proliferation test was able to detect a type IV hypersensitivity in the blood [64–66] by measuring activated T-helper lymphocytes under food allergen stimulation with flow-cytometry [66]. In 49 of 54 AFR dogs this test accurately provided positive reactions against one or more food allergens [66], however this test is not commercially available at this time.

AD in animals is diagnosed by history, clinical examination and exclusion of all differential diagnoses. Positive reactions are frequently seen in healthy dogs on both intradermal tests [67] and serum tests for allergen-specific IgE [68]. The total serum IgE concentrations seem to have no clinical relevance in the dog [44]. Once AD is diagnosed in an animal, testing can be used in combination with clinical historical information to choose which allergens should be selected for allergen immunotherapy. Serum tests for allergen-specific IgE and intradermal tests are equally useful and both are still performed with allergen extracts in animals, in contrast to component-resolved tests such as single molecule CAP testing or ImmunoCAP ISAC 112 microarray in human medicine [45]. Prick puncture testing is not performed routinely in veterinary medicine, as intradermal testing is an established and safe diagnostic tool with a very low risk of adverse effects [69].

### Treatment of atopic dermatitis in small animals

Therapy selection depends on the pet’s condition, especially the severity of the lesions and degree of pruritus and owner preference and especially in cats—on the ability to medicate. The therapy needs to be reassessed regularly and adapted to the individual [70]. With the exception of avoidance of the causative allergen [71], in general there are two different treatment approaches: specific with allergen immunotherapy or symptomatic with a variety of drugs. The combination of various drugs can increase the chance of remission [70].

### Specific allergen-targeted therapy

Allergen immunotherapy (AIT) is the only possibly curative treatment option [70]. In approximately 50–75% of the atopic animals desensitization is effective [72–76]. In those animals, it is often recommended to continue the treatment lifelong [70, 77]. In contrast to human medicine where accelerated immunotherapy (“rush”) is only advised in selected patients, due to the high frequency of systemic adverse reactions, in dogs rush-immunotherapy is effective and safe with no reported increased risk of adverse reactions [76, 78, 79]. Intralymphatic desensitization (ILIT) in humans was reported to reduce the therapeutic interval from 3 years to 8 weeks with less severe adverse effects [80]. ILIT is also used in veterinary medicine, but with less predictable success than in humans and a recent report showed the need for ongoing immunotherapy at regular intervals [81]. Sublingual immunotherapy (SLIT) was introduced to veterinary medicine some years ago, but so far limited published data is available [82].

### Biologicals

Monoclonal antibodies are a focus of research in human medicine. They target specific receptors or cytokines and are highly specific and effective in blocking their target molecule. Lokivetmab is a monoclonal caninised anti-IL-31 antibody, that was recently approved for the use in atopic dogs. It significantly decreased pruritus for at least 4 weeks [83]. Its efficacy is comparable to oral prednisolone. Lokivetmab is regarded as safe without any immediate hypersensitivity reactions. Adverse reactions were similar in dogs treated with lokivetmab to those treated with placebo [84]. In the treatment group, 2.5% of the dogs produced antibodies against lokivetmab [84] but their clinical significance is unclear at this point. To date no other therapeutic monoclonal antibody exists in veterinary medicine.

### General anti-inflammatory and anti-pruritic treatment

In severely affected dogs and cats, glucocorticoids, ciclosporin, oclacitinib or lokivetmab are used for symptomatic therapy due to their clinical efficacy and high success rates of 70–80% [85].

*Glucocorticoids* are inexpensive, universally available and have been the mainstay of treatment for allergic pets for many years. However, the potentially severe adverse effects of oral and particularly injectable depot glucocorticoids such as polyuria and polydipsia, polyphagia, muscle atrophy, secondary skin infections, calcinosis cutis and others have led to the development of alternative drugs for dogs and cats.

*Ciclosporin*, a calcineurin inhibitor, is highly effective in dogs and cats with comparable results to glucocorticoids [86–88]. The initial daily dosage can be reduced in the majority of animals to every other day or twice weekly [86, 87]. Mild gastrointestinal symptoms (e.g. diarrhoea and vomiting) frequently occur at the beginning of treatment but usually resolve during continued administration [89]. Hirsutism, gingival hyperplasia and hyperplastic dermatitis are reported adverse effects which typically resolve with dose reduction or discontinuation [87]. Sporadic case reports exist of immunologically naive cats newly infected with *Toxoplasma gondii*, developing systemic and even fatal clinical signs [90, 91]. It is recommended to evaluate anti-toxoplasma antibodies in outdoor cats and cats fed raw meat prior to initiating cyclosporine therapy.

*Oclacitinib* is a selective inhibitor of janus kinase 1. Janus kinase 1 is involved in the signaling pathways of the receptors for IL-2, IL-4, IL-6, IL-13 and IL-31 [92], and thus aims at blocking the Th2 pathway. It is administered to dogs at a dose of 0.4–0.6 mg/kg twice daily for 2 weeks and then daily at that dose is reported to be as effective as glucocorticoids [93, 94]. In comparison to cyclosporine, oclacitinib has a more rapid effect and gastrointestinal adverse effects are less frequently observed [95]. Skin infections and histiocytomas were reported with increased frequency in dogs on longer term oclacitinib therapy [93]. Oclacitinib given to a small number of cats with atopy-like dermatitis over a 4 week period was effective [96], however the dose required was higher than for dogs, the period of monitoring was short and both more and larger studies are needed before it can be recommended as standard therapy.

Different *antihistamines* are associated anecdotally with individual responses, therefore a trial therapy with various antihistamines over 7–14 days is recommended [97, 98]. Histamine binds to four receptor subtypes (H1to H4) which are expressed in different tissues [99]. Its interaction with the high-affinity H1 receptor is known to cause cutaneous vasodilatation, oedema, and wheal formation. Histamine can also attract effector cells such as eosinophils to the region of inflammation [99]. Antihistamines targeting the cutaneous H1 receptors block the binding of histamine and are used most frequently in order to reduce the pruritus in atopic dogs [100]. Antihistamines binding to the H4 receptor showed an anti-inflammatory and anti-pruritic effect in mice [101, 102]. However, they did not prevent the development of acute skin lesions in a canine atopic model [103]. A double blinded, placebo-controlled, cross-over study evaluated the efficacy of dimetindene and a combination of hydroxyzine and chlorpheniramine in 19 atopic dogs and concluded that in both groups a limited, but significant improvement on pruritus was achieved, nevertheless other drugs might additionally be needed [104]. Many owners consider antihistamines useful therapeutic agents for their pets’ allergy [105]. The recommended dosage of antihistamines is much higher in cats and dogs than in humans. Dogs can rapidly metabolise hydroxyzine to cetirizine and need twice daily hydroxyzine orally at 2.0 mg/kg [99]. In one study a positive effect of antihistamines, mainly loratadine and cetirizine, was shown in 67% of 31 atopic cats [33]. In contrast, in another study, cats with allergic dermatitis treated with cetirizine hydrochloride showed no significant differences in lesion- and pruritus-scores to those treated with placebo [106].

A future non-specific treatment alternative might be the subcutaneous injection of cytosine-phosphate guanine *oligodeoxynucleotides* bound to gelatine nanoparticles (CpG GNPs). This therapy resulted in decreased lesions and pruritus in ≥ 50% of atopic dogs, similar to what is seen with AIT and the mRNA expression of IL-4 was also decreased in those dogs [107]. However, this treatment is currently not commercially available.

Due to their hair coat and compliance issues, *topical treatment* of dogs and cats can be difficult for owners and therefore it is less frequently used than in humans [44]. Topical glucocorticoid ointments can be used for localised skin lesions in sparsely haired areas, but prolonged application may result in skin atrophy [98]. Topical hydrocortisone aceponate was effective for canine AD [108, 109] and feline atopy-like dermatitis [110]. Topical calcineurin inhibitors such as tacrolimus have been used successfully in localized lesions of canine AD [111, 112]. Atopic dogs may benefit from shampoo therapy [113, 114].

Adding *dietary supplementations* such as essential fatty acids (EFA), probiotics or vitamins can have a positive benefit for atopic animals. EFA are used to treat AD in cats [115] and dogs [116]. Oral EFA can improve the coat quality, strengthen the skin barrier and reduce the transepidermal water loss [117]. Moreover EFA can lower the amount of glucocorticoids and cyclosporine needed to control clinical signs of canine AD [118, 119].

*Probiotics* are microorganisms that are claimed to provide health benefits when consumed [120, 121]. Their mechanism is not completely elucidated, but may involve binding Toll-like receptors and downregulate the allergic predominately TH2-mediated response [122, 123]. *Lactobacillus paracasei K71* given orally to atopic dogs led only to a slight improvement of lesion- and pruritus-score [124]. However, the medication score was reduced significantly indicating a potential benefit as a complementary therapy [124]. *Lactobacillus rhamnosus GG* given to puppies led to a reduction of immunologic indicators of AD, even though no significant clinical improvement was observed [125].

In human studies a positive impact of *cholecalciferol* on AD was detected [126–128]. Similarly, systemic cholecalciferol reduced pruritus and lesion scores in dogs with AD [129].

## How to diagnose and manage AD in the difficult animal and its owner

Both diagnosis and therapy of AD in cats and dogs requires patience, time and effort. An appropriate diagnostic work-up will ensure the correct diagnosis of the disease and concurrent flare factors and usually includes an elimination diet and ectoparasite control as well as cutaneous cytology to rule out secondary infections. It is not uncommon for dogs and cats with environmental allergies to be affected by flea bite hypersensitivity or AFR concurrently [32, 50] and it can be difficult to determine how much of the symptomatology is due to which type of antigen. In those animals, the diagnostic work-up may require an elimination diet with several provocation trials and an extensive flea control in addition to repeated examinations of the animal in order to ensure adequate resolution of secondary infections and concurrent flea bite hypersensitivity. Many owners do not believe that their dog or cats’ problem is food triggered and are reluctant to limit their pet’s food intake to one protein and one carbohydrate source. AFR is not necessarily related to a recent diet change and in one report most of the dogs with AFR received the same food for 2 years or longer before symptoms arose [130]. An elimination diet with restriction to one food source in outdoor or free-roaming cats, dogs living on a farm or in a household with small children is difficult to impossible. Cats should ideally be kept inside for the diet period [131] and some dogs need to wear a muzzle during walks to prevent the rapid gobbling down of potentially allergenic food stuff [51, 132]. Throughout the diagnostic process owner noncompliance can be an issue, because of high costs, continuous drug administration and the organisational and emotional problems associated with feeding a limited elimination diet. Thorough and repeated client education and support contribute to good owner compliance [133]. A diary for the owners to record the daily pruritus, drug side effects or pitfalls during the elimination diet can increase their motivation [131]. Low palatability, refusal of the diet (particularly in cats) or gastrointestinal symptoms such as diarrhoea or constipation can decrease compliance [134]. A gradual change to the “new” food can minimise those problems. In contrast to dogs it is not an option to allow cats to “starve for a few days” while offering the new diet, as a negative energy balance due to anorexia can initiate hepatic lipidosis [135]. Owners may need to be made aware of the “traps” of an elimination diet [131], for example tooth paste and medications for pets are frequently flavoured with animal proteins and thus will interfere with the elimination diet. Chewable drugs or drugs in gelatin capsules need to be avoided [131] as it was shown that dogs allergic to corn and soy showed cutaneous flares after receiving chewable capsules containing pig protein, soy and milbemycin [132]. Similarly many owners do not consider treats “food” and rely on those for dog training. Those treats need to be replaced with one made of the protein used in the diet to optimise outcome. Secondary infections, most often *Malassezia* spp. in dogs [117, 136] and staphylococci in dogs and cats [137–140] may mimic the clinical signs of allergy and require investigation of other possible causes for the infection. After establishing the diagnosis, it is important to explain to the owner that an allergy is a lifelong disease and thus will usually require lifelong management. Multiple adaptations of therapy may be needed depending on the individual animal’s condition and flare factors. Treatment options, their costs, efficacy and safety need to be discussed with the owners in detail. Some may prefer a rapid clinical improvement with a potent systemic drug, whereas others may not want to risk this drug’s side effects. Short-term relief can lead to a higher owner compliance. The emotional relationship between owner and animal should not be underestimated. Often owners suffer with their animal and sleepless nights of the owners are the consequence of a highly pruritic animal.

## Unmet needs and research

At this point, the pathogenesis of AD in dogs and cats is not fully elucidated. Multiple genes are implicated [14]. However, further genomic studies and investigations on breed differences may allow a better understanding of the heritability. Research on the role of CD25+ FoxP3+ T cells is ongoing [20]. In human medicine the hygiene hypothesis ascribes the increasing allergy risk to a modern environment and life style with less pathogen exposure [141, 142]. This might apply to animals in the same way as the prevalence of AD seems to be lower in dogs living in rural areas [143]. More studies are needed to evaluate environmental influence on AD in dogs and cats, possibly enabling prophylactic measures in the future. Allergen-specific IgE can be measured, but a correlation of the results with clinical signs is not always present [144]. Multiple serum allergy tests are offered, but cannot be used to diagnose AD. Additionally, inter- and intralaboratory variability of some of those tests is high [145–148]. With regard to treatment for AD the first monoclonal antibody for atopic dogs, an anti-IL-31-antibody, is available with promising clinical results, but the consequences of a long-term blockade of IL-31 are unknown at this point [84]. Individual phenotypes of AD in dogs and cats may respond better to specific drugs than others. More studies and pooling of data to obtain numbers to achieve significance are needed to evaluate the efficacy of specific drugs in specific breeds and pheno- as well as genotypes to allow tailored patient-oriented therapy in veterinary medicine. AIT is typically administered via subcutaneous injections in both dogs and cats, there is however a lack of well-powered dose-finding studies in animals. Further and comparative studies are also needed to investigate which alternative application route is most suitable in which clinical situation. Using recombinant allergens such as *Dermatophagoides farinae* allergen (Der f 2) [149, 150] may result in more reproducible results and a higher success rate compared to standard AIT and ILIT [151]. Modified allergen preparations such as allergoids, allergen peptides as well as alteration with adjuvants may decrease the risk of adverse effects and increase efficacy [152]. First studies evaluated bacterial oligodeoxynucleotides in canine AD [79, 107] with promising results.

## Conclusion

AD in pets is diagnosed by history, clinical signs and the ruling out of differential diagnoses. Allergy tests (intradermal tests and serum tests for allergen-specific IgE) cannot be used as a diagnostic tool for AD, but rather in association with clinical history permit the selection of relevant allergens for immunotherapy. Multiple flare factors such as additional flea-bite hypersensitivity and AFR and secondary bacterial or yeast infections can complicate AD in the dog and cat and need to be identified, prevented and/or treated. Intensive and regular communication with the pet owner and a diagnostic work-up and treatment tailored to the individual pet and owner’s needs is essential for a good compliance and optimal outcome.

## Abbreviations

AD: atopic dermatitis
Ig: immunoglobulin
IL: interleukin
Th2: T helper 2
PO: periostin
AFR: adverse food reaction
AIT: allergen immunotherapy
ILIT: intralmyphatic immunotherapy
SLIT: sublingual immunotherapy
EFA: essential fatty acids
CpG GNPs: cytosine-phosphateguanine oligodeoxynucleotides bound to gelatine nanoparticles
Der f 2: *Dermatophagoides farinae* allergen
